# Colon cancer-derived myofibroblasts increase endothelial cell migration by glucocorticoid-sensitive secretion of a pro-migratory factor

**DOI:** 10.1016/j.vph.2016.10.004

**Published:** 2017-02

**Authors:** Zuzanna Drebert, Mark MacAskill, Dahlia Doughty-Shenton, Karolien De Bosscher, Marc Bracke, Patrick W.F. Hadoke, Ilse M. Beck

**Affiliations:** a Laboratory of Experimental Cancer Research, Department of Radiation Oncology & Experimental Cancer Research, Ghent University, Ghent, Belgium; b Cancer Research Institute Ghent (CRIG), Ghent, Belgium; c University/BHF Centre for Cardiovascular Science, The Queen's Medical Research Institute, University of Edinburgh, Edinburgh, United Kingdom; d Edinburgh Phenotypic Assay Centre, The Queen's Medical Research Institute, University of Edinburgh, Edinburgh, United Kingdom; e Receptor Research Laboratories, Nuclear Receptor Lab (NRL), VIB Department of Medical Protein Research, Ghent University, Ghent, Belgium

**Keywords:** Myofibroblast, Angiogenesis, Endothelial cell, Glucocorticoid, Cancer

## Abstract

Angiogenesis is important in cancer progression and can be influenced by tumor-associated myofibroblasts. We addressed the hypothesis that glucocorticoids indirectly affect angiogenesis by altering the release of pro-angiogenic factors from colon cancer-derived myofibroblasts.

Our study shows that glucocorticoids reduced prostanoids, urokinase-type plasminogen activator (uPA) and angiopoietin-like protein-2 (ANGPTL2) levels, but increased angiogenin (ANG) in supernatant from human CT5.3hTERT colon cancer-derived myofibroblasts. Conditioned medium from solvent- (CMS) and dexamethasone (Dex)-treated (CMD) myofibroblasts increased human umbilical vein endothelial cell (HUVEC) proliferation, but did not affect expression of pro-angiogenic factors or tube-like structure formation (by HUVECs or human aortic ECs). In a HUVEC scratch assay CMS-induced acceleration of wound healing was blunted by CMD treatment. Moreover, CMS-induced neovessel growth in mouse aortic rings *ex vivo* was also blunted using CMD. The latter effect could be ascribed to both Dex-driven reduction of secreted factors and potential residual Dex present in CMD (indicated using a dexamethasone-spiked CMS control). A similar control in the scratch assay, however, revealed that altered levels of factors in the CMD, and not potential residual Dex, were responsible for decreased wound closure.

In conclusion, our results suggest that glucocorticoids indirectly alter endothelial cell function during tumor development *in vivo*.

## Graphical abstract

**Figure:**
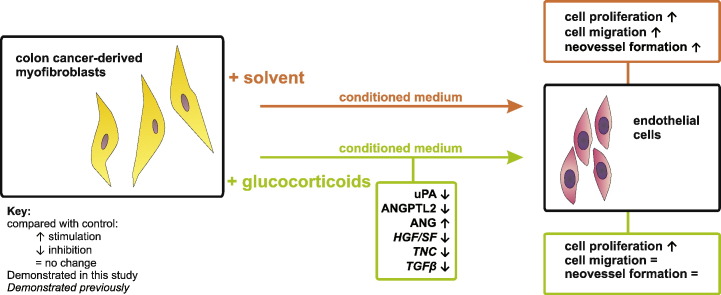

## 1 Introduction

Angiogenesis, the formation of new blood vessels from an existing vascular network [1], is essential for embryonic growth. In healthy adults angiogenesis is restricted to discrete physiological processes (e.g. the regulation of the reproductive tract, muscle growth) and contributes to wound healing [2]. Excessive or impaired angiogenesis has also been implicated in disease pathogenesis (e.g. in malignant or inflammatory disorders [2]), and is associated with promotion of tumor growth and metastasis. Consequently, the potential of angiogenesis as a therapeutic target (e.g. in cancer [1], [2], [3], retinopathy [4] and tissue ischemia [5]) has attracted considerable research interest.

Tumors use blood vessels not only as a source of nutrients and oxygen, but also to transport cancer cells to establish a new, metastatic site [6]. Cancer cells can directly modulate angiogenesis via secretion of pro-angiogenic factors, such as vascular endothelial growth factor (VEGF), angiopoietins, basic fibroblast growth factor (bFGF), interleukins (ILs) or transforming growth factors (TGFs) [3], [7]. Epithelial tumors consist of cancer cells and a surrounding microenvironment composed of an extracellular matrix, stromal cells, inflammatory cells and endothelial cells (ECs). All these components play an important role during tumor development [8]. Cancer-associated fibroblasts (myofibroblasts) are present at the invasive edge of the tumor and share properties of both smooth-muscle cells and fibroblasts. Myofibroblasts, which are essential during wound healing and embryonic development [9], can also influence tumor progression [10], [11] either directly, through paracrine signaling to cancer cells, or indirectly, by modulation of protease activity, modulation of extracellular matrix remodeling, and recruitment of immune cells [8], [10]. Myofibroblasts also have the potential to alter EC function and influence tumor angiogenesis [8], [11]. In breast cancer, cancer-associated fibroblasts promote vascularization by recruiting endothelial progenitor cells to the tumor via increased release of stromal-cell derived factor 1 (SDF-1) [12]. Moreover, prostaglandin (PG)E_2_-stimulated intestinal sub-epithelial myofibroblasts display an increased expression of vascular endothelial growth factor (VEGF) and hepatocyte growth factor/scatter factor (HGF/SF), which promote EC migration [13].

Glucocorticoids (GCs) are steroidal ligands of the glucocorticoid receptor (GR), which belongs to the nuclear receptor superfamily. Stimulation of GR regulates many physiological processes, mainly via gene transactivation or transrepression [14]. Consequently, glucocorticoids are clinically important as potent anti-inflammatory compounds in treatment of autoimmune diseases [15], and as adjuvants in cancer therapy [16]. Moreover, GCs provide an effective treatment of infantile hemangiomas (IHs) [17]. GC-mediated inhibition of angiogenesis is well-documented [18] and has therapeutic potential in the treatment of cancer [19], [20]. The direct, growth-inhibitory influence of GCs on vascular smooth muscle cells is well-established [21], [22]. Furthermore, more recent investigations have demonstrated GR-dependent, GC-mediated inhibition of tube-like structure formation by ECs *in vitro*, independent of GCs' anti-inflammatory actions [23]. GCs can also inhibit angiogenesis indirectly by suppression of pro-angiogenic factors, such as VEGF and IL-8, produced by prostate cancer cells [20], and possibly by extracellular matrix degradation or modification of cytokine production [24].

We recently reported that GCs regulate myofibroblasts, decreasing production and secretion of a number of factors linked to cancer progression and invasion: tenascin C (TNC), TGFβ, HGF/SF [25], [26], [27]. These factors are all known to also affect the angiogenic response through a number of mechanisms [28], [29], [30]. Combined with our data, these studies suggest that GCs could have the ability to inhibit myofibroblast-induced stimulation of angiogenesis by altering the composition of the myofibroblast secretome. Therefore, this investigation addressed the hypothesis that exposure of colon cancer-derived myofibroblasts to GCs can reduce secretion of angiogenic factors and thus inhibit their ability to promote pro-angiogenic changes in ECs.

## 2 Materials and methods

### 2.1 Cells and reagents

Human stromal colon cancer-derived myofibroblasts (CT5.3hTERT cells) were isolated as described [26], [31] and cultured (37 °C, 10% CO_2_) in Dulbecco's modified Eagles Medium (DMEM; Life Technologies, Merelbeke, Belgium) supplemented with 10% fetal calf serum (Greiner bio-one, Wemmel, Belgium), 100 U/ml penicillin and 0.1 mg/ml streptomycin (Life Technologies). Primary human umbilical vein endothelial cells (HUVEC; Promocell, Heidelberg, Germany) and human aortic endothelial cells (HAoEC; Promocell) were cultured in Endothelial Cell Growth Medium-2 (EGM2; Lonza, Wokingham, UK), containing all manufacturer-supplied supplements (2% FCS, 0.1% VEGF, 0.4% hFGF-2, 0.1% R^3^-IGF-1, 0.1% hEGF, 0.1% ascorbic acid, 0.1% heparin, 0.1% GA-100) except hydrocortisone. HUVECs were cultured (37 °C, 5% CO_2_) on 0.1% gelatin-coated flasks and were studied between passages 2 and 7. In experiments we used EGM2 containing 2% FCS or 0% FCS, abbreviated respectively EGM2^S +^ and EGM2^S −^.

Dexamethasone (Dex), hydrocortisone (Hcrt), prednisolone (Pred), fluocinolone acetonide (FA) and the GR antagonist RU38486 (RU) were purchased from Sigma-Aldrich (Diegem, Belgium). All reagents were dissolved in ethanol and used at a final concentration of 1 μM, except RU (2 μM). A selective GR modulator (SEGRM), compound A (CpdA) was prepared as previously described [32] and used at a final concentration of 10 μM. The total solvent concentration (maximally 0.1%) was consistent in all conditions.

### 2.2 Conditioned medium preparation

Conditioned medium (CM) was obtained from 10 × 10^6^ CT5.3hTERT myofibroblasts and prepared as described [26]. Briefly, cells were washed three times with serum-free DMEM and treated for 48 h with solvent (ethanol), Dex (1 μM), Hcrt (1 μM), Pred (1 μM), CpdA (10 μM) or RU (2 μM) in serum-free DMEM. After this incubation CM was collected, concentrated 10-fold using centrifugal filter tubes with a 3 kDa cut-off (Amicon Ultra, Merck Millipore, Darmstadt, Germany), filter-sterilized (0.2 μm pore size) and stored (− 20 °C) for subsequent functional and biochemical assays. For functional assays CM from solvent and Dex-treated myofibroblasts (CMS and CMD, respectively) were diluted with EGM2^S +^ or EGM2^S −^ or with serum-free DMEM prior to treatment. Taking into account the concentrating procedure of CM and further dilution in the functional assays, the maximal final concentration of Dex in the CMD treatment was calculated to be 50 nM. CM concentrations and dilutions used in particular experiments are listed in Supplementary Table 1.

### 2.3 Protein analysis: protein array, Western blot and immunoassay (ELISA)

CM from CT5.3hTERT myofibroblasts treated with Dex or solvent (CMS and CMD, respectively) were collected after 48 h, 4-fold concentrated and subjected to Ray Bio® Biotin Label-based Human Antibody Array I (Raybiotech, GA, USA, cat no: AAH-BLM-I-2) which allows simultaneous analysis of expression levels of 507 human target proteins (including cytokines, chemokines, adipokines, growth factors, angiogenic factors, proteases, soluble receptors and soluble adhesion molecules) in cell culture supernatants. The assay was performed according to the manufacturer's instructions with the results visualized using X-Ray films (GE Healthcare, Diegem, Belgium) and the signal evaluated using ImageJ software [33]. For further analysis, we set the threshold value for the ratio between relative protein signals in CMS vs. CMD as > 1.5. Selected factors analyzed using the protein array are listed in Supplementary Table 2.

For further validation of the protein array results, CT5.3hTERT myofibroblasts were incubated for 48 h with steroids (Dex, Hcrt, Pred; 1 μM), CpdA (10 μM), RU (2 μM) or solvent. Conditioned media were collected, concentrated (10-fold) and protein concentrations were evaluated using the Lowry method [34]. Samples were prepared in SDS sample buffer (50 mM Tris pH 6.8; 2% SDS; 10% glycerol; bromophenol blue; 100 mM DTT), loaded (25 μg) onto an SDS-PAGE gel and subjected to the standard Western blot protocol, as described by Santa Cruz (Santa Cruz, Heidelberg, Germany). The proteins were probed using the following primary anti-human antibodies: anti-uPA (H-140) (1/500, Santa Cruz Biotechnology, cat no: sc-14019), anti-ANG I (H-123) (1/500, Santa Cruz Biotechnology, cat no: sc-9044) and anti-ANGPTL2 (P-13) (1/500, Santa Cruz Biotechnology, cat no: sc-107143). Results were visualized using species-specific HRP-linked secondary antibodies and reagents: anti-rabbit (1/4000, GE Healthcare, cat no: NA934V), anti-goat (1/3000, Santa Cruz Biotechnology, cat no: sc-2020), ECL solution (Thermo Scientific, Gent, Belgium) and X-Ray films (GE Healthcare). Signal quantifications were performed using ImageJ software [33].

The internalization and subsequent degradation of the acetylated low density lipoprotein (Ac-LDL) is a characteristic feature of endothelial cells. In order to evaluate whether the conditioned medium from myofibroblasts affects the basic endothelial character of HUVECs, we performed an Ac-LDL uptake assay. Briefly HUVECs were incubated for 24 h in EGM2^S +^ (control), DMEM, CMS or CMD. DMEM and 10-fold concentrated CM were diluted 1:1 with EGM2^S +^. An Ac-LDL assay was then performed, as described (see Supplementary methods in Supporting Information).

In order to determine the concentrations of prostanoids in conditioned medium from myofibroblasts and HUVECs, and in HUVEC lysates, we performed immunoassays (ELISAs) for prostaglandin F_2α_ (PGF_2α_), prostacyclin (PGI_2_; by assessing 6-keto-PGF_1α_) and prostaglandin E_2_ (PGE_2_), according to manufacturer's instructions (Enzo Life Sciences, Antwerp, Belgium, cat no: ADI-900-069, ADI-900-001 and ADI-900-004, respectively). Absorbance was quantified on Paradigm Detection Platform (Beckman Coulter) using SoftMax Pro 6.1 software. HUVEC lysates were prepared from cells treated with EGM2^S +^ (control), CMS or CMD (diluted 1:1 with EGM2^S +^, giving a final 5-fold concentration of CM). After 24 h cells were lyzed with TOTEX buffer (20 mM Hepes/KOH pH 7.9; 0.35 M NaCl; 20% glycerol; 1% NP40; 1 mM MgCl_2_; 0.5 mM EDTA; 0.1 mM EGTA; 1/100 HALT Protease and Phosphatase Inhibitor Cocktail, ThermoFisher Scientific, cat no: 78440) and the lysates were subjected to immunoassays.

### 2.4 RNA isolation and RT-qPCR

CT5.3hTERT myofibroblasts were incubated for 48 h with steroids (Dex, Hcrt, FA, Pred; 1 μM), CpdA (10 μM), RU (2 μM) or solvent (control). HUVECs were incubated for 24 h with EGM2^S +^ (control), DMEM, CMS or CMD. DMEM and CM were diluted 1:1 with EGM2^S +^, the final CM concentration was 5-fold. To isolate the total RNA from myofibroblasts we used TRIzol reagent (Life Technologies) and to isolate HUVEC RNA we used an RNeasy Kit (Qiagen, Hilden, Germany), according to the manufacturer's instructions. Reverse transcription (RT) of myofibroblast RNA was performed using the iScript kit (Bio-Rad, Temse, Belgium), whilst RT of HUVEC RNA was performed using QuantiTect Reverse Transcription Kit (Qiagen). The cDNA obtained was subjected to quantitative PCR (qPCR) using LightCycler 480 SYBR Green I Master reagents (Roche Diagnostics, Rotkreuz, Switzerland), according to the manufacturer's instructions. qPCR reactions were performed in triplicate using the LightCycler 480 system (Roche Diagnostics), with the following conditions: (A) initial denaturation 95 °C, 5 min; (B) 45 cycles of denaturation 95 °C, 15 s, annealing and elongation 60 °C, 45 s. Primer sequences are listed in Supplementary Table 3. Specific signal of the gene of interest was normalized to the respective geometric mean expression level of 3 reference genes (GAPDH, PPIB, 36B4).

### 2.5 Cell viability (MTT) and proliferation (SRB) assays

To test viability HUVECs were seeded in 96-well plates, equilibrated in EGM2^S +^ for 24 h and incubated for 24 h with DMEM, CMS or CMD. DMEM and 10-fold concentrated CM were diluted 1:1 with EGM2^S +^. As a negative control HUVECs were treated with 10% Triton X-100 (Sigma-Aldrich) for 1 h (data not shown). Cell viability was assessed using a classic 3-(4,5-dimethylthiazol-2-yl)-2,5-diphenyltetrazolium bromide (MTT) assay [35], performed with reagents purchased from Sigma-Aldrich.

Proliferation was assessed using a sulforhodamine-B (SRB) test, as described previously [36]. HUVECs seeded in 96-well plates were left to equilibrate in EGM2^S +^ for 24 h and then incubated in EGM2^S +^ or EGM2^S −^, DMEM, CMS, CMS + Dex (50 nM) or CMD for 24-72 h. DMEM and 10-fold concentrated CM were diluted 1:1 with EGM2^S +^ or EGM2^S −^. Results were obtained using a Molecular Devices OPTImax Microplate Reader and the SoftMax® Pro 3.0 software. Data were expressed on a scale where maximal proliferation in controls (EGM2^S +^ at 72 h) was set to 100%.

### 2.6 Scratch assay

HUVEC migration was assessed using the IncuCyte ZOOM Scratch assay (Essen Bioscience, Hertfordshire, UK) according to manufacturer's instructions. Briefly, 3 × 10^4^ HUVECs/well were seeded in 96-well culture plates and cultured for 18 h in EGM2^S +^ at 37 °C, 5% CO_2_. A scratch was then made using the WoundMaker tool (Essen Bioscience). The cells were washed twice with medium, and the medium was then replaced with EGM2^S +^, EGM2^S −^, CMS, CMS + Dex (50 nM) or CMD. 10-Fold concentrated CM were diluted 1:1 with EGM2^S −^. Plates were then installed in the IncuCyte ZOOM system and images (10 × magnification) of the wound were recorded in each well every hour for 48 h. Scratch closure rate was evaluated with the IncuCyte software, expressed as percentage of relative wound density (RWD) over a 30 h period. RWD = 0 at time 0 and 100% when cell confluence within the wound area is equal to that outside the initial wound area, thus normalizing for changes in cell density due to proliferation or other non-motogenic pharmacological effects. The area under the curve (AUC) was calculated for each condition and the results are expressed as AUC from RWD.

### 2.7 Tube-like structure (TLS) formation assay

The TLS assay was performed by seeding HUVECs or HAoECs onto Matrigel™, as previously described [37]. Briefly, HUVECs and HAoECs (15 × 10^3^ cells/well) were seeded in 96-well plates coated with Matrigel™ matrix (Corning, Flintshire, UK) in either EGM2^S +^, DMEM, CMS or CMD. DMEM and 10-fold concentrated CM were diluted 1:4 with EGM2^S +^. This assay required a lower concentration of CM than that used in other experiments (4:1 ratio EGM2^S +^:CM, giving a final concentration of 2 × basal CM) as ECs failed to generate TLS networks when EGM2^S +^ was used in 1:1 ratio with DMEM. Phase-contrast images (5 × magnification) of the centre of each well were taken 3 h, 6 h and 23 h post induction and TLS formation was evaluated using the Angiogenesis Analyzer plug-in developed for the ImageJ software [33] by Carpentier et al. [38]. The total length of tubes, number of junctions, and number of segments were calculated from images taken when the network reached stability (6 h post induction for HUVECs; 3 h post induction for HAoECs).

### 2.8 Aortic ring assay

For the ex vivo aortic ring assay [39] C57BL/6 male mice aged 8–12 weeks (Charles River Laboratories) were sacrificed by CO_2_ asphyxiation at day 0 and the thoracic aortas were isolated and washed with serum-free DMEM. Isolated aortas were cleaned of connective tissue, divided into 1–2 mm rings and embedded in rat tail collagen type 1 (1 mg/ml, Sigma-Aldrich). Rings were then incubated (37 °C, 5% CO_2_) in serum-free DMEM (control), CMS, CMS + Dex (50 nM) or CMD. 10-fold concentrated CM were diluted 1:1 with serum-free DMEM. Media were replaced after 3 and 7 days in culture. Phase-contrast microscopy was used to count outgrowths on days 5, 7 and 10. Phase-contrast images (5 × magnification) were taken at the corresponding time points. Sprout lengths were measured on pictures obtained after 10 days post treatment using ImageJ software [33]. Higher power images of formed sprouts are displayed in Supplementary Fig. 6.

### 2.9 Statistical analyses

Data are presented as mean ± standard deviation or as a Tukey's box plot (Suppl. Fig. 2). Statistical analysis was performed using GraphPad Prism 5.03 with a one-way analysis of variance (ANOVA) and Tukey's multiple comparisons post-test, or with Mann-Whitney *U* test, as appropriate. The applied test is indicated in the figure legends. A p < 0.05 was considered statistically significant. Where applicable, results were expressed as a relative number and the untreated condition was set as 1, 100 or 100% and other conditions were recalculated accordingly.

## 3 Results

### 3.1 Glucocorticoids modify secretion of angiogenic factors by myofibroblasts

**Fig. 1:**
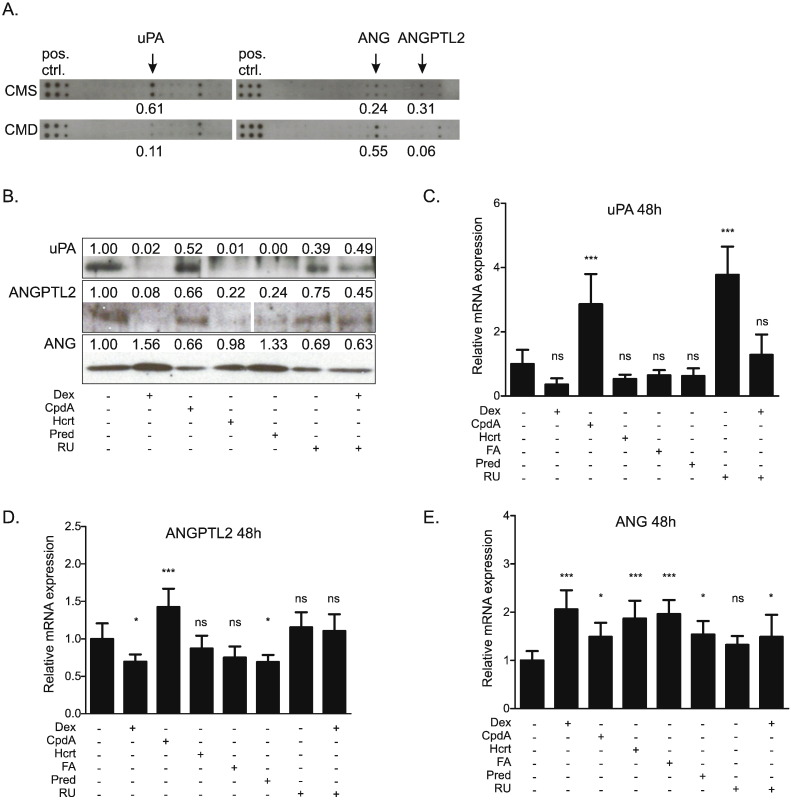
Glucocorticoids alter the secretion of proteins implicated in angiogenesis from cultured myofibroblasts. (A) CT5.3hTERT cells were treated with solvent or Dex (1 μM). After 48 h cell supernatants were collected, 4-fold concentrated and subjected to Ray Bio® Biotin Label-based Human Antibody Array I. Relevant fragments of the array are displayed. The six dots displayed on the left of the array act as a positive control (pos.ctrl.). (B) CT5.3hTERT cells were treated with solvent, Dex (1 μM), CpdA (10 μM), Hcrt (1 μM), Pred (1 μM), or RU (2 μM) or co-treated with Dex (1 μM) and RU (2 μM) for 48 h. Cell supernatants were collected, 10-fold concentrated and subjected to Western blot analysis for the detection of uPA, ANG and ANGPTL2. Protein bands representing ANGPTL2 belong to the same blot. Results are representative of three independent experiments. (A, B) Western blot and protein array signals were quantified using ImageJ software [33]. (C, D, E) CT5.3hTERT cells were treated for 48 h with solvent, Dex (1 μM), CpdA (10 μM), Hcrt (1 μM), FA (1 μM), Pred (1 μM), RU (2 μM) or co-treated with Dex (1 μM) and RU (2 μM). mRNA isolated from cells was subjected to RT-qPCR assaying uPA, ANG and ANGPTL2 mRNA levels. Results were normalized to the respective geometric mean of GAPDH, PPIB and 36B4 reference genes' mRNA levels. Results are shown as the mean ± SD of three independent experiments and statistical analysis was performed using a one-way analysis of variance (ANOVA) and Tukey's multiple comparisons post-test. ns: not significant, *: p < 0.05, ***: p < 0.001.

In order to obtain a broader insight into the effects of GR modulation on colon cancer-derived myofibroblasts, we performed a protein array which detects over 500 different proteins from cell supernatants. Analysis of the protein array data (Fig. 1A) indicated that incubation with Dex (1 μM) for 48 h reduced the expression of urokinase-type plasminogen activator (uPA) and angiopoietin-like protein-2 (ANGPTL2), but increased expression of angiogenin (ANG) in supernatant from CT5.3hTERT myofibroblasts. The array also identified a number of factors present in the CM from myofibroblasts that were not sensitive to Dex treatment. Selected angiogenesis-related and inflammatory factors are listed in the Supplementary Table 2.

Western blot analyses (Fig. 1B) and RT-qPCRs (Fig. 1C–E), were used to verify the results obtained from the protein array and to possibly extend our findings to other GR ligands and modulators. Western blot analysis of 10-fold concentrated cell supernatants confirmed that Dex (1 μM; 48 h) reduced protein levels of uPA, and ANGPTL2, whilst increasing ANG protein levels (Fig. 1B). A similar regulation pattern was seen with other glucocorticoids (Hcrt and Pred). In contrast, the SEGRM CpdA (10 μM; 48 h) did not reduce uPA and ANGPTL2 protein levels and did not upregulate ANG. RU (2 μM; 48 h) alone had no effect, but blocked Dex-induced changes which suggests a GR-regulated mechanism. RT-qPCR of mRNA isolated from CT5.3hTERT cells showed that glucocorticoids seemed to reduce expression of uPA (Fig. 1C) and ANGPTL2 (Fig. 1D), but this only achieved significance for the effects of Dex and Pred on ANGPTL2. The length of exposure to Dex matters here, as Dex-induced reduction of uPA expression was found to be significant after a shorter (6 h) exposure (Supplementary Fig. 1). Consistently, all glucocorticoids significantly upregulated ANG (Fig. 1E). In contrast, to what is observed for protein, CpdA yielded different results at the transcriptional level, following 48 h of treatment, and upregulated the mRNA levels of uPA, ANGPTL2 and ANG. Treatment with RU had no effect on ANG and ANGPTL2 mRNA levels, but dramatically increased expression of uPA. Any effect of Dex was lost or reduced in the presence of RU (Fig. 1C–E).

### 3.2 Conditioned medium from dexamethasone-treated myofibroblasts contains decreased levels of prostanoids

**Fig. 2:**
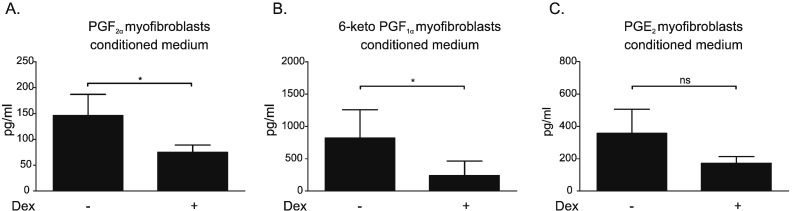
Conditioned medium from dexamethasone-treated myofibroblasts contains reduced levels of prostanoids. CT5.3hTERT cells were treated with solvent or Dex (1 μM). After 48 h cell supernatants were collected, 10-fold concentrated and analyzed (ELISA) for (A) PGF_2α_, (B) PGI_2_ (by assessing 6-keto-PGF_1α_) and (C) PGE_2_ levels. Results are the mean ± SD of four independent experiments and statistical analysis was performed using a one-way analysis of variance (ANOVA) and Tukey's multiple comparisons post-test, ns: not significant, *: p < 0.05.

Prostanoids are known to influence cell proliferation and migration. Immunoassays (ELISAs) demonstrated that PGF_2α_, PGI_2_ (by assessing 6-keto-PGF_1α_) and PGE_2_ were all present in conditioned medium from solvent-treated myofibroblasts (CMS) (Fig. 2). There was a pattern of decreased levels of all three prostanoids in conditioned medium from myofibroblasts exposed to dexamethasone (CMD), which achieved significance for PGF_2α_ (Fig. 2A) and PGI_2_ (Fig. 2B) but not for PGE_2_ (Fig. 2C).

Immunoassays (ELISA) demonstrated that PGF_2α_, PGI_2_ and PGE_2_ were also present in medium from HUVECs (Supplementary Fig. 2A–C). Exposure to CMS (24 h) did not alter the concentration of these prostanoids in HUVEC supernatants and this response was not altered if the conditioned medium was derived from Dex-treated myofibroblasts (CMD). These prostanoids were also detected in HUVEC lysates but their concentrations were not altered by 24 h exposure to CMS or CMD (Supplementary Fig. 2D–F).

### 3.3 HUVEC proliferation is promoted by conditioned medium from myofibroblasts

The impact of conditioned medium from myofibroblasts on the basic endothelial character of HUVECs was assessed using an Ac-LDL uptake assay. Results indicate Ac-LDL uptake was not altered in HUVECs following 24 h exposure to DMEM, CMS or CMD (Supplementary Fig. 3).

**Fig. 3:**
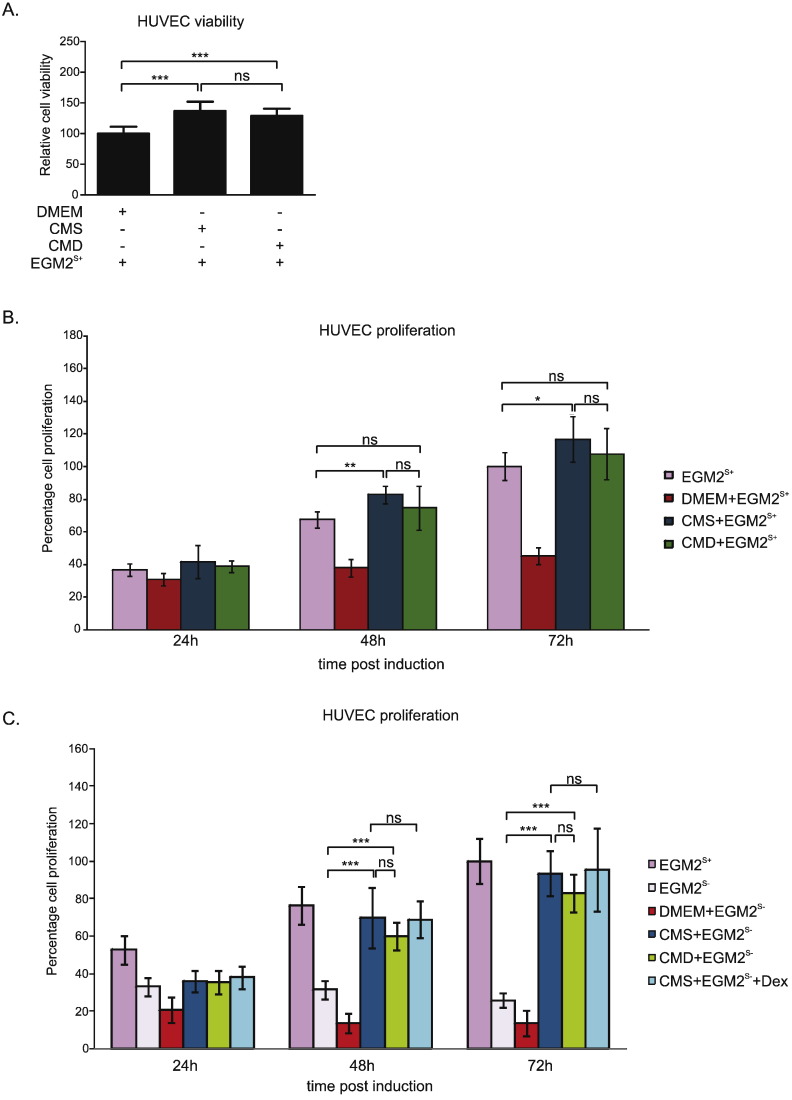
HUVEC proliferation is promoted by conditioned medium from myofibroblasts. (A) HUVECs were treated with EGM2^S +^ mixtures with DMEM, CMS or CMD in 1:1 ratio. After 24 h cells were subjected to MTT assay and percentage cell viability was assessed. Obtained values were normalized to the values obtained from cells treated with DMEM and other conditions were recalculated accordingly. (B) HUVECs were treated with either EGM2^S +^ (control) or with EGM2^S +^ mixtures with DMEM, CMS or CMD in 1:1 ratio. After 24 h, 48 h and 72 h cells were subjected to SRB assay and percentage cell proliferation was calculated. Obtained values were normalized to a control of untreated cells at 72 h, which indicates their maximal proliferation. (C) HUVECs were treated with either EGM2^S +^, EGM2^S −^ or with EGM2^S −^ mixtures with DMEM, CMS, CMD or CMS + Dex (50 nM) in 1:1 ratio with EGM2^S −^. Obtained values were normalized to a control of cells treated with EGM2^S +^ at 72 h, which indicates their maximal proliferation. Results (A, B, C) are the mean ± SD of at least three independent experiments and statistical analysis was performed using a one-way analysis of variance (ANOVA) and Tukey's multiple comparisons post-test. ns: not significant, *: p < 0.05, **: p < 0.01, ***: p < 0.001.

Prior to proliferation experiments, an MTT assay was performed and demonstrated no negative effect on viability or metabolism from either treatment (Fig. 3A). On the contrary, CMS and CMD treatment increased the production of the MTT formazan product.

In order to investigate the impact of myofibroblast CM on EC growth we performed an SRB assay. In comparison with the control treatment (EGM2^S +^) HUVEC proliferation was reduced by exposure to DMEM (Fig. 3B). However, proliferation was increased compared with EGM2^S +^ when the HUVECs were exposed to CMS after 48 and 72 h. Use of CMD did not result in a significant difference with the EGM2^S +^ control.

In the absence of FCS (Fig. 3C), both CMS and CMD induced a dramatic increase in HUVEC proliferation compared with EGM2^S −^ control, at 24 h and 72 h post treatment. Addition of Dex (50 nM) to CMS did not alter HUVEC proliferation.

### 3.4 Conditioned medium from solvent-treated myofibroblasts causes an increase in HUVEC migration which is lost with conditioned medium from Dex-treated cells

**Fig. 4:**
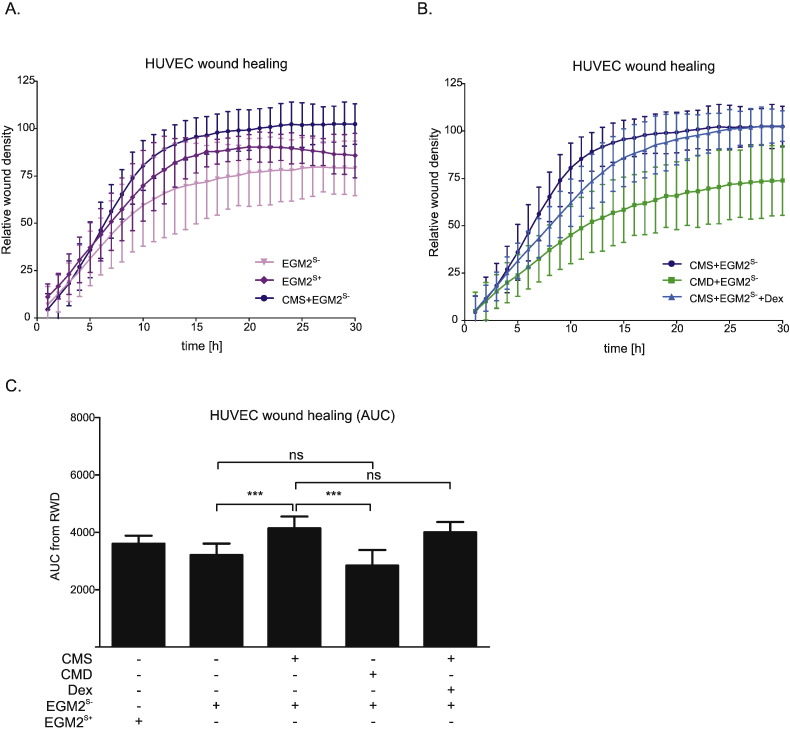
Acceleration of HUVEC migration by myofibroblast conditioned medium is lost when the myofibroblasts are exposed to dexamethasone. (A, B, C) HUVECs were cultured in EGM2^S +^. After 18 h a wound was created in the confluent cell monolayer. Cells were washed and treated with either EGM2^S +^, EGM2^S −^, or EGM2^S −^ mixtures with CMS, CMD or CMS + Dex (50 nM) in 1:1 ratio. (A, B) The wound healing process was examined with the IncuCyte ZOOM system, measuring percentage relative wound density (RWD) for each condition every hour. (C) Area under curve (AUC) was calculated for each treatment and displayed in parallel. Results (A, B, C) are represented as the mean ± SD of four independent experiments and statistical analysis was performed using a one-way analysis of variance (ANOVA) and Tukey's multiple comparisons post-test. ns: not significant, ***: p < 0.001.

One of the crucial events of angiogenesis includes EC migration into perivascular stroma, due to the presence of pro-angiogenic factors. In the scratch assay (Fig. 4, Supplementary Fig. 4), 30 h exposure to CMS increased (by approximately 25% compared with EGM^S −^) the area under the curve (AUC) (Fig. 3C), indicating accelerated wound healing. This acceleration was not seen when cells were exposed to CMD (Fig. 4B, C). These data suggest, therefore, that CMS contains a factor(s) that stimulate HUVEC migration which is not present in CMD. Direct addition of Dex (50 nM) did not abolish the CMS-induced increase in wound healing (Fig. 4B, C), indicating the lack of effects with CMD was not due to residual Dex.

### 3.5 Conditioned medium from myofibroblasts does not influence tube-like structure formation by HUVECs or HAoECs

**Fig. 5:**
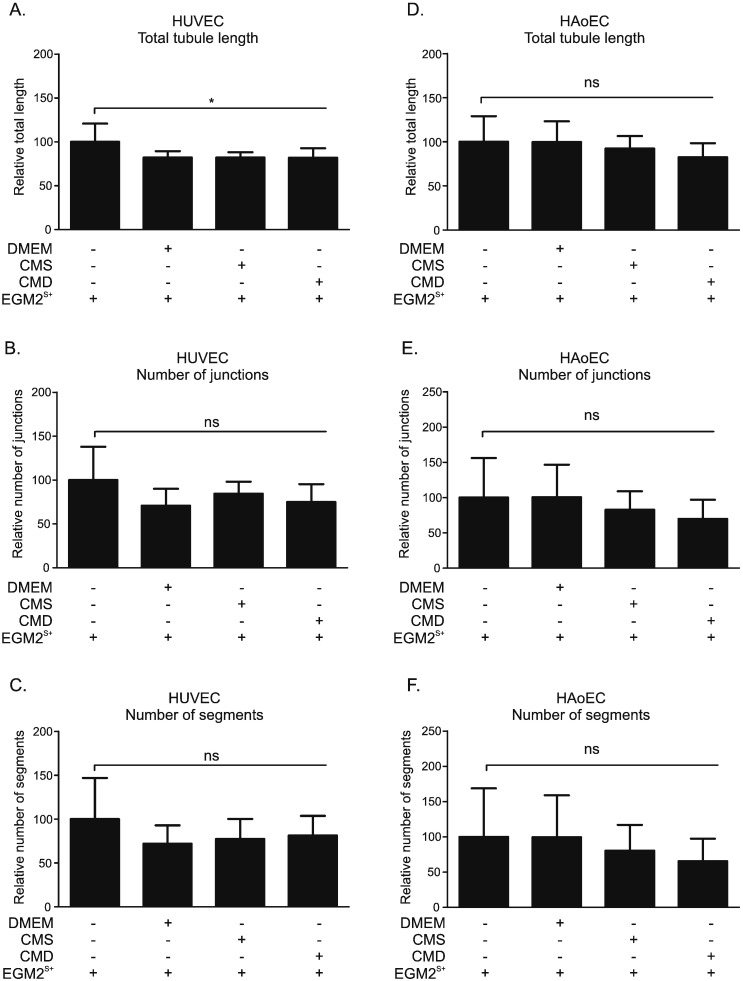
Conditioned medium from myofibroblasts does not affect tube-like structure formation by HUVECs or HAoECs. HUVECs (A, B, C) and HAoECs (D, E, F) were seeded on Matrigel-coated wells and treated with either EGM2^S +^ or EGM2^S +^ mixtures with DMEM, CMS or CMD in 1:4 ratio. Phase-contrast images were taken at 6 h post induction for HUVECs and 3 h post induction for HAoECs. The total tubule length (A, D), number of junctions (B, E) and number of segments (C, F) were assessed using Angiogenesis Analyzer plug-in for ImageJ software [33], [38]. Results are the mean ± SD of three independent experiments and statistical analysis was performed using a one-way analysis of variance (ANOVA) and Tukey's multiple comparisons post-test, ns: not significant, *: p < 0.05.

The ability of ECs to form three-dimensional structures (tube-like structures, TLS) represents cell differentiation belonging to a later phase of angiogenesis (Supplementary Fig. 5). The ability of HUVECs to form a net of TLS was mildly impaired after incubation with DMEM, CMS or CMD diluted in a ratio of 1:4 with EGM2^S +^, as compared to untreated control (EGM2^S +^). This inhibition only achieved significance for total tubule length (Fig. 5A), but not for number of junctions (Fig. 5B) or number of segments (Fig. 5C). Exposure to conditioned media had a similar effect in HAoECs with neither CMS nor CMD significantly altering total tubule length (Fig. 5D), number of junctions (Fig. 5E) or number of segments (Fig. 5F).

### 3.6 Conditioned medium from myofibroblasts alters gene expression in HUVECS

**Fig. 6:**
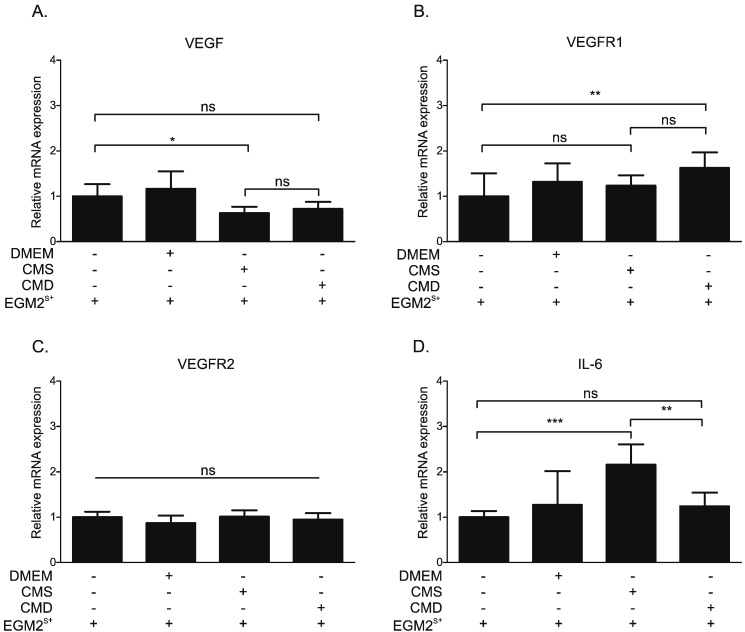
Influence of conditioned medium from myofibroblasts on angiogenesis-related gene expression in HUVECs. (A, B, C, D) HUVECs were treated with either EGM2^S +^ or EGM2^S +^ mixtures with DMEM, CMS or CMD in 1:1 ratio. After 24 h, mRNA isolated from cells was subjected to RT-qPCR assaying (A) VEGF, (B) VEGFR1, (C) VEGFR2 and (D) IL-6 mRNA levels. Obtained results were normalized to the respective geometric mean of GAPDH, PPIB and 36B4 reference genes' mRNA levels. Results are the mean ± SD of three independent experiments and statistical analysis was performed using a one-way analysis of variance (ANOVA) and Tukey's multiple comparisons post-test. Ns: not significant, *: p < 0.05, **: p < 0.01, ***: p < 0.001.

In order to investigate whether the CM from myofibroblasts affects the expression of angiogenesis-related genes in HUVECs, we performed RT-qPCR for VEGF, VEGFR1, VEGFR2 and IL-6. RT-qPCR was run on mRNA obtained from HUVECs exposed for 24 h to DMEM or conditioned medium, diluted in a 1:1 ratio with EGM2^S +^. Exposure to CM produced an apparent reduction in VEGF mRNA expression (Fig. 6A) that achieved significance (0.62-fold change) only for the comparison of CMS with the untreated (EGM2^S +^) control. CM had little effect on VEGFR1 (Fig. 6B) or VEGFR2 (Fig. 6C) mRNA, with a small increase (1.63 fold change) in VEGFR1 seen only when CMD-treated cells were compared with untreated (EGM2^S +^) controls. In contrast, exposure to CMS induced a clear (2.16-fold) increase in IL-6 transcripts (Fig. 6D) which was not observed when cells were exposed to CMD.

### 3.7 Conditioned medium from myofibroblasts promotes outgrowth formation from mouse aortic rings

**Fig. 7:**
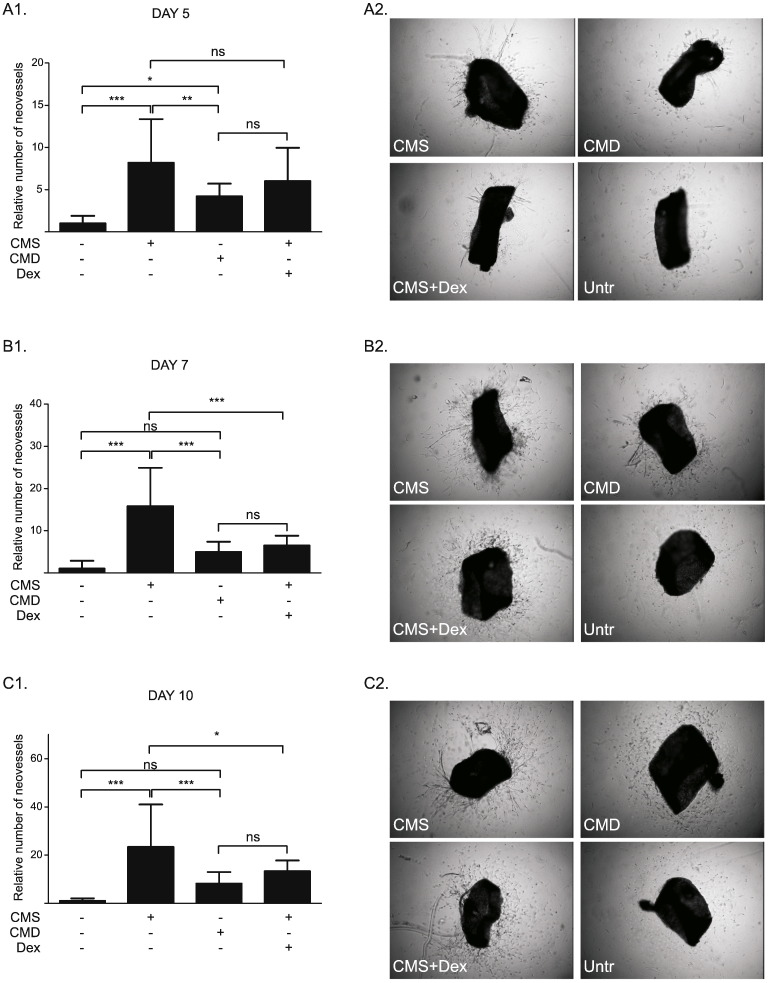
Conditioned medium from myofibroblasts promotes neovessel formation in mouse aortic rings embedded in collagen. (A, B, C) Explants were prepared from aortas isolated from adult male C57BL/6 mice. After embedding in collagen, aortic rings were cultured in serum-free DMEM (control) or treated with CMS, CMD or CMS + Dex (50 nM), in 1:1 ratio with serum-free DMEM. Vascular sprouts were quantified after 5 days (A1, A2), 7 days (B1, B2) and 10 days (C1, C2) in culture. Left panel histograms represent the mean ± SD of six independent experiments. Results were analyzed using a one-way analysis of variance (ANOVA) and Tukey's multiple comparisons post-test. ns: not significant, *: p < 0.05, **: p < 0.01, ***: p < 0.001.

**Fig. 8:**
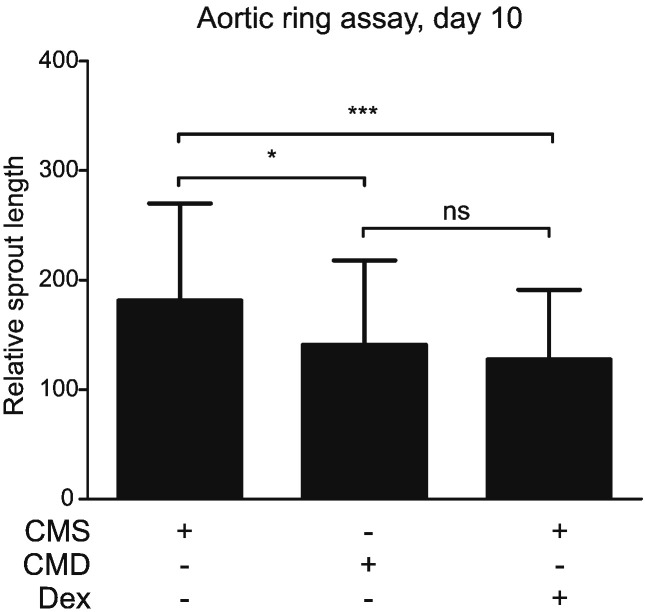
Conditioned medium from myofibroblasts increases outgrowth length in mouse aortic rings embedded in collagen. Explants were prepared from aortas isolated from adult male C57BL/6 mice. After embedding in collagen, aortic rings were treated with CMS, CMD or CMS + Dex (50 nM), in a 1:1 ratio with serum-free DMEM. Images of explants and vascular sprouts were captured after 10 days and measurement of outgrowth length was performed using ImageJ software [33]. Results are the mean ± SD of six independent experiments and were analyzed using a one-way analysis of variance (ANOVA) and Tukey's multiple comparisons post-test. ns: not significant, *: p < 0.05, ***: p < 0.001.

The organ culture-aortic ring assay enabled investigation of the effects of myofibroblast conditioned medium in a more complex model of angiogenic tube formation, involving the presence of non-endothelial cells (smooth muscle cells, fibroblasts, pericytes, inflammatory cells) in an intact arterial ring (rather than in 2 dimensional culture). Ex vivo outgrowth vessel formation in mouse aortic rings (Fig. 7) was increased by exposure to CMS after 5 days (Fig. 7A), 7 days (Fig. 7B) and 10 days (Fig. 7C) of incubation, compared to untreated control (DMEM). This effect was less pronounced using CMD and by addition of Dex (50 nM) to CMS. In addition, the length of outgrowths (Supplementary Fig. 6) was reduced by exposure to CMD or by addition of Dex (50 nM) to CMS (Fig. 8).

## 4 Discussion

This investigation addressed the hypothesis that exposure of tumor-derived myofibroblasts to glucocorticoids would reduce secretion of angiogenic factors and inhibit their ability to promote angiogenesis. We showed that conditioned medium from colon cancer-derived myofibroblasts stimulated proliferation and migration of HUVECs. Secretion of certain angiogenic factors was altered in conditioned medium from Dex-treated myofibroblasts (CMD), and this was associated with a reduced HUVEC migration, but did not affect HUVEC proliferation. Exposure to conditioned media only slightly altered expression of angiogenic genes in HUVECs, and had no effect on tube-like structure formation in a 2-dimensional assay (with HUVECS and HAoECs). In contrast, in an *ex vivo* model (mouse aorta), conditioned media from myofibroblasts increased the number and length of vascular outgrowths. This effect was impaired when myofibroblasts had been exposed to Dex; most likely a combined result of direct inhibition by residual steroid in the conditioned medium together with Dex-driven reduction of certain factors secreted by myofibroblasts.

Angiogenesis is a complex, multi-step process regulated by a balance between pro- and anti-angiogenic factors [1], [3]. It can be modified at various stages, including degradation of basement membrane and EC shape change, invasion, migration and proliferation of ECs to form a migrating column, EC differentiation, formation of tight connections and capillary tubes, fusion with other vessels and cell maturation and pruning [40]. Stromal myofibroblasts have the potential to regulate angiogenesis during tumor development. They are recruited by cancer cells and act as potent promoters of tumor growth and invasion [10]. For example, breast cancer-associated fibroblasts promote tumor microvascularization, leading to enhanced tumor growth [12]. Myofibroblast-mediated regulation of vessel formation in cancer could be attributed to direct and/or indirect modulation of angiogenesis [8], [10]. We have previously shown the GC-sensitive and GR-regulated release of several pro-angiogenic factors (TNC, TGFβ and HGF/SF) by cultured colon cancer-derived myofibroblasts [25]. The present study extends this work by showing that these cells secrete factors that promote survival, proliferation and migration of ECs. In culture conditions devoid of serum, CM from colon cancer-derived myofibroblasts could compensate for the absence of FCS, thus maintaining HUVEC proliferation (Fig. 3C) and migration (Fig. 4A). A similar increase in HUVEC migration was demonstrated with CM collected from intestinal sub-epithelial 18Co myofibroblasts, an effect that was stimulated by pre-treatment of the myofibroblasts with prostaglandin (PG)E_2_[13].

Since myofibroblastic CM regulated migration and proliferation of ECs, it was reasonable to propose that it might also influence angiogenesis through direct interaction with the endothelium. However, the failure of CM to stimulate TLS formation by HUVECs or HAoECs in a 2-dimensional assay suggests that this was not the case (Fig. 5). Furthermore, a lack of effect on angiogenesis is consistent with the relatively small impact of CM on expression of angiogenic factors in HUVECs (Fig. 6). VEGF is a key promoter of angiogenesis, and acts through interaction with VEGFR2 [3], [41]. VEGFR1, which has a 10-fold weaker kinase activity than VEGFR2, may act as a VEGF-trap and, thus, suppress angiogenesis [41], [42]. IL-6 is a multifunctional pro-inflammatory cytokine which has potent pro-angiogenic properties [43], [44]. Interestingly, CM from intestinal subepithelial 18Co myofibroblasts were also ineffective unless activated with PGE_2,_ which increased VEGF expression in those myofibroblasts [13]. Similarly, mouse embryonic fibroblasts did not promote tube formation unless pre-treated with CM from gastric tumor cells, which increased expression of VEGF in the fibroblasts [45]. These findings suggest that CM obtained from colon-cancer myofibroblasts contain insufficient levels of VEGF and, coupled with a lack of VEGF activation in HUVECs treated with CM, could explain the inability of CM to stimulate tube formation by isolated HUVECs and HAoECs.

GCs are exploited clinically, predominantly for their anti-inflammatory properties, for the treatment of numerous disorders, including asthma and rheumatoid arthritis [15]. They also serve as adjuvants in tumor therapy [16]. However, the influence of GCs on the solid tumor and its microenvironment is controversial and not fully understood [46]. In prostate and breast cancer GC therapy has some benefits, whereas in gastro-intestinal cancer GC treatment has no effect and in lung cancer may even be detrimental [47]. We have previously shown that production and secretion of TNC, TGFβ and HGF/SF by myofibroblasts is reduced by GC treatment [25]. This is comparable with the demonstration here that GCs reduce expression and secretion of uPA and ANGPTL2, whilst upregulating ANG (Fig. 1). This response is probably mediated via a GR-dependent mechanism, since Dex is relatively GR selective and its effects were blocked by GR antagonism with RU. The alterations in uPA, ANGPTL2 and ANG secretion were observed with other GCs, namely Hcrt, FA and Pred. Although the non-steroidal SEGRM CpdA [32] is able to transrepress the expression of several GR-regulated genes in myofibroblasts [25], it suppresses neither uPA nor ANGPTL2 protein levels. As previously reported [25], [32] CpdA is unable to transactivate GC-inducible genes via a classic GRE-mediated mechanism. Therefore, consistent with our results, any effect on ANG protein or mRNA level was not expected. These findings support a common mechanism for the action of GCs and add to the evidence that CpdA has different signaling properties than classic GCs [25]. The factors identified as affected by GCs have been linked to cancer progression and/or angiogenesis. uPA regulates vascular remodeling [48] and its expression correlates with tumor angiogenesis and tumor vessel invasion in gastric and breast cancer [49], [50]. ANG is named for its ability to stimulate vessel growth, in normal and pathological states, including in tumors [51]. Angiopoietin-like proteins can stimulate vascular cells and influence metabolism and tumor biology [52]. Thus it was logical to predict that modulation of these factors by exposure of the myofibroblasts to Dex might influence angiogenesis. Moreover, levels of prostaglandins, factors known to modulate inflammatory response and to promote cancer progression [53], [54], were also reduced by Dex treatment in myofibroblast conditioned medium (Fig. 2). This corresponds with previous reports listing glucocorticoids as inhibitors of prostaglandin synthesis [55].

The failure of CMD to alter the CMS-induced stimulation of HUVEC proliferation (Fig. 2B, C) indicates that modulation of uPA, ANGPTL2, ANG or prostaglandins does not influence growth of these cells. There was no evidence that myofibroblast conditioned media induce prostaglandin production by HUVECs. This contrasted with the dramatic reduction in HUVEC migration when CMD was compared with CMS (Fig. 3B, C). The fact that this effect was not replicated by direct addition of Dex to CMS indicates that the reduced migration is the result of changes in the myofibroblastic secretome. In support, some of the components of CM that are suppressed by GC treatment can influence cell motility. uPA release from TGFβ-stimulated endometrial stromal cells increases migration of human microvascular ECs [56], whilst knock-down of the ANGPTL2 gene impairs migration of endothelial colony forming cells [57]. The same is true for HGF/SF and tenascin C, which we have previously shown to be downregulated by GCs [25]. HGF/SF has well-known mitogenic and motogenic actions on ECs [58], [59] and, thus, a reduction in HGF/SF could explain impaired HUVEC migration by HUVECs. Tenascin C promotes EC migration by binding to annexin II on the target cell surface, as well as by promoting phosphorylation of focal adhesion kinase [60], [61]. Thus, decreased levels of these proteins in CM from Dex-treated myofibroblasts are likely to explain the impaired motogenic response seen in HUVECs. Prostaglandins, especially PGE_2_ and PGI_2_, are known pro-angiogenic factors that directly induce EC survival, migration and tube-formation by activating respective receptors [54], [62]. Therefore, insufficient levels of these factors in CMD may have also negatively influenced HUVEC migration.

It was notable that, in contrast to the 2-dimensional assay with HUVECs and HAoECs (Fig. 5), CMS did increase the number and length of vascular outgrowth formation in mouse aortic explants cultured *ex vivo* (Fig. 7, Fig. 8). This is unlikely to be simply due to a functional difference between umbilical vein and aortic ECs as single cultures of HUVECs and HAoECs responded in a similar way to CM in the TLS assay. Outgrowth formation in this assay is dependent on growth factor release from adventitial inflammatory cells [63]. Concomitant herewith, it is notable that the most dramatic change in transcript expression in HUVECs treated with CM was a 2-fold increase in IL-6 (Fig. 6D), a pro-inflammatory cytokine that can influence angiogenesis. IL-6 and indeed many inflammatory proteins were not detected in the myofibroblast conditioned medium (Supplementary Table S2). It has been reported that IL-6 stimulates angiogenesis directly leading to increased proliferation and migration of ECs [44], as well as endothelial progenitor cells [43]. This suggests the presence of inflammation-stimulating molecules in the myofibroblastic secretome that are also sensitive to down-regulation by GCs. These results suggest that CM from myofibroblasts increases angiogenesis indirectly by stimulation of growth factor release by other (non-endothelial) cells in the vascular wall. The reduced effect observed with CMD can be attributed to both Dex-driven reduction of certain factors from myofibroblastic secretome and residual Dex in the medium, as addition of a comparable concentration of Dex to CMS had a similar effect, but slightly less pronounced (Fig. 7). This is consistent with the well-documented direct angiostatic properties of GCs [21], [22], [23], [39], including suppression of outgrowth formation in the aortic ring assay [39].

In conclusion, this investigation has demonstrated that colon cancer-derived myofibroblasts secrete pro-angiogenic factors and stimulate endothelial cell migration. This migration is inhibited by exposure of the myofibroblasts to GCs which alter the components of the myofibroblast secretome. A similar modulation of angiogenesis appears to be the result of indirect interaction of CM with non-endothelial vascular cells, possibly through activation of vascular inflammatory pathways. This work suggests that treatment with GCs may reduce the ability of cancer-derived myofibroblasts to stimulate endothelial cell migration and angiogenesis, through both direct and indirect effects on the vascular wall.

The following are the supplementary data related to this article.

Supplementary Figure 1. Expression of uPA in conditioned medium is reduced when the myofibroblasts are exposed for 6h to dexamethasone.
Supplementary Figure 2. Levels of prostanoids produced in HUVECs are not affected by treatment with conditioned medium from myofibroblasts.
Supplementary Figure 3. Conditioned media does not alter acetylated LDL uptake by HUVECs.
Supplementary Figure 4. Representative images of scratch wound assay. Image 2

Supplementary Figure 5 Representative images of tube-like structure formation assay. Supplementary Figure 5.

Supplementary Figure 6 Representative higher-power images of the aortic rings and outgrowths. Supplementary Figure 6.

Supplementary material comprises of supplementary methods, supplementary tables and legends for supplementary figures. Image 3
