# Supplementary figures and images for: Colon cancer-derived myofibroblasts increase endothelial cell migration by glucocorticoid-sensitive secretion of a pro-migratory factor
Source: Vascul Pharmacol. 2017 Feb;89:19–30. doi: 10.1016/j.vph.2016.10.004 (PMC5328197; doi:10.1016/j.vph.2016.10.004)

Drebert\_Supplementary Figure 1

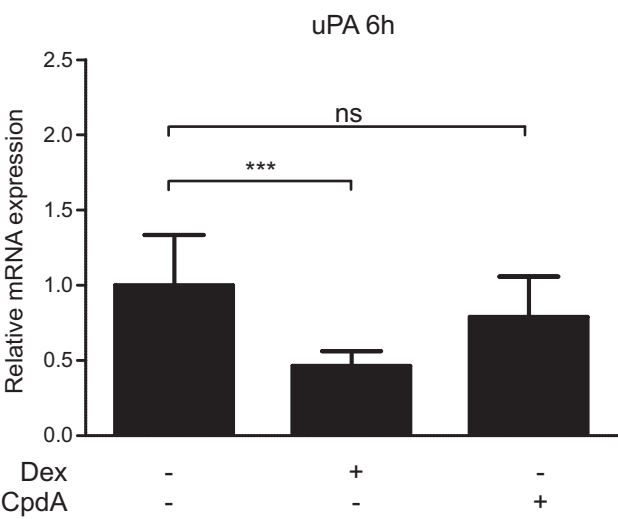

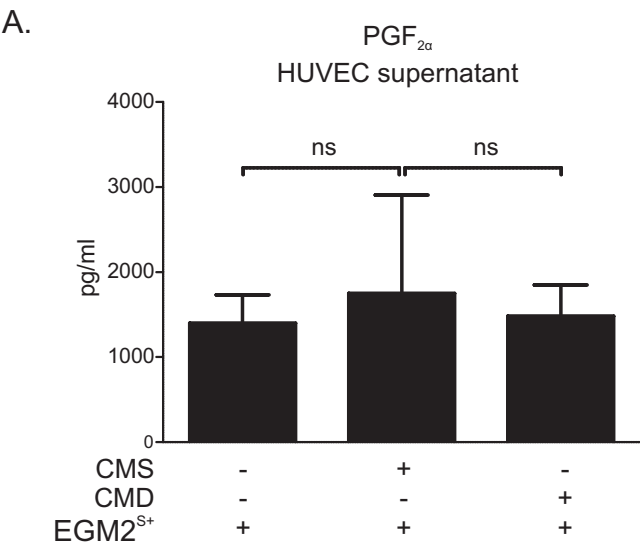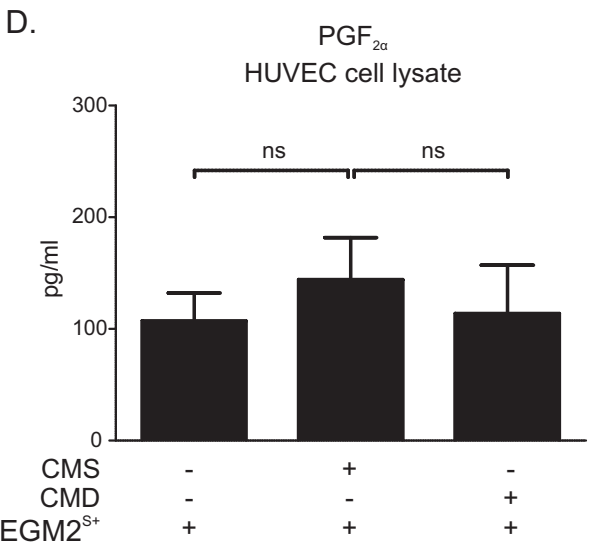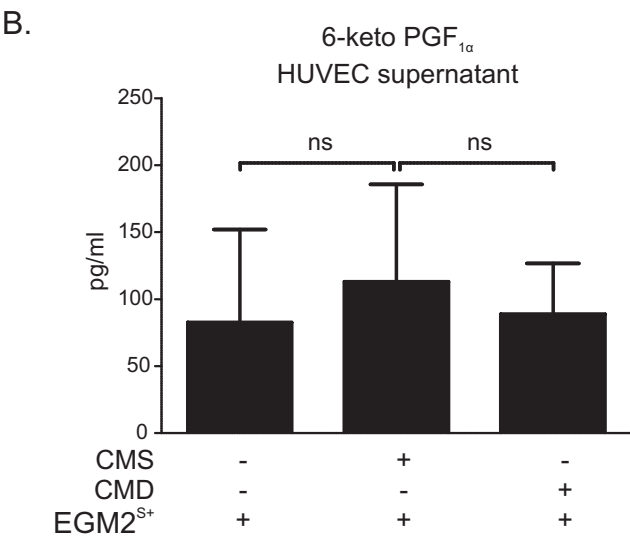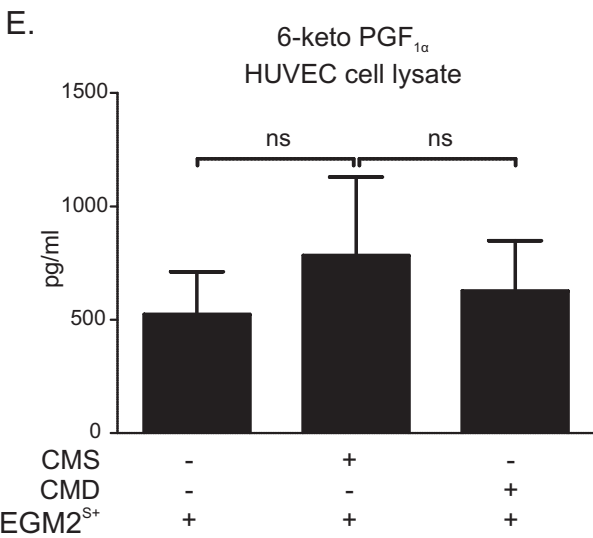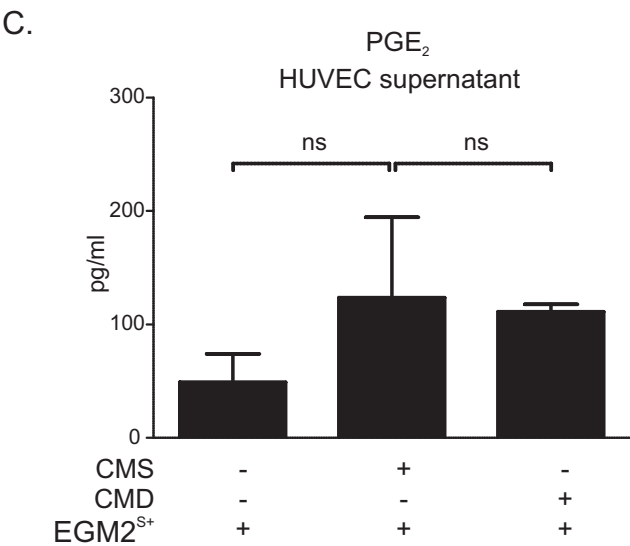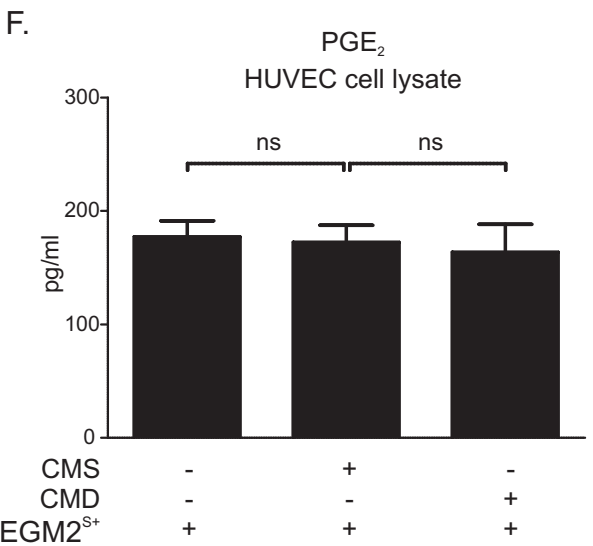

Drebert\_Supplementary\_Figure 3

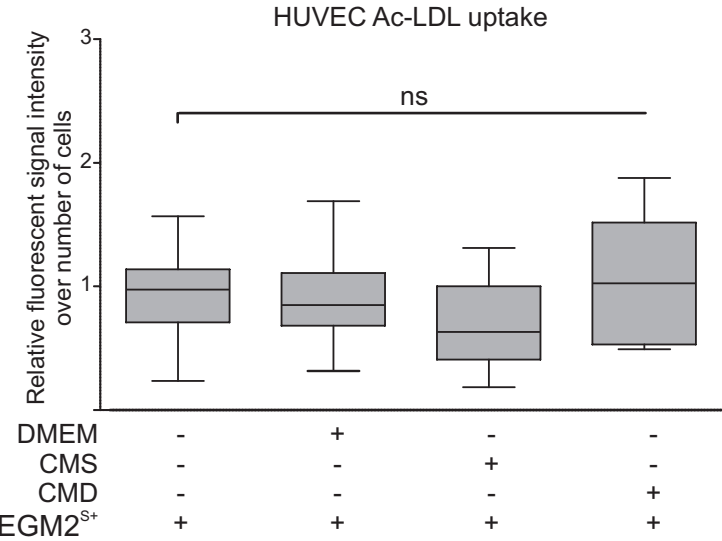

ZD\_Supplementary Figure 4

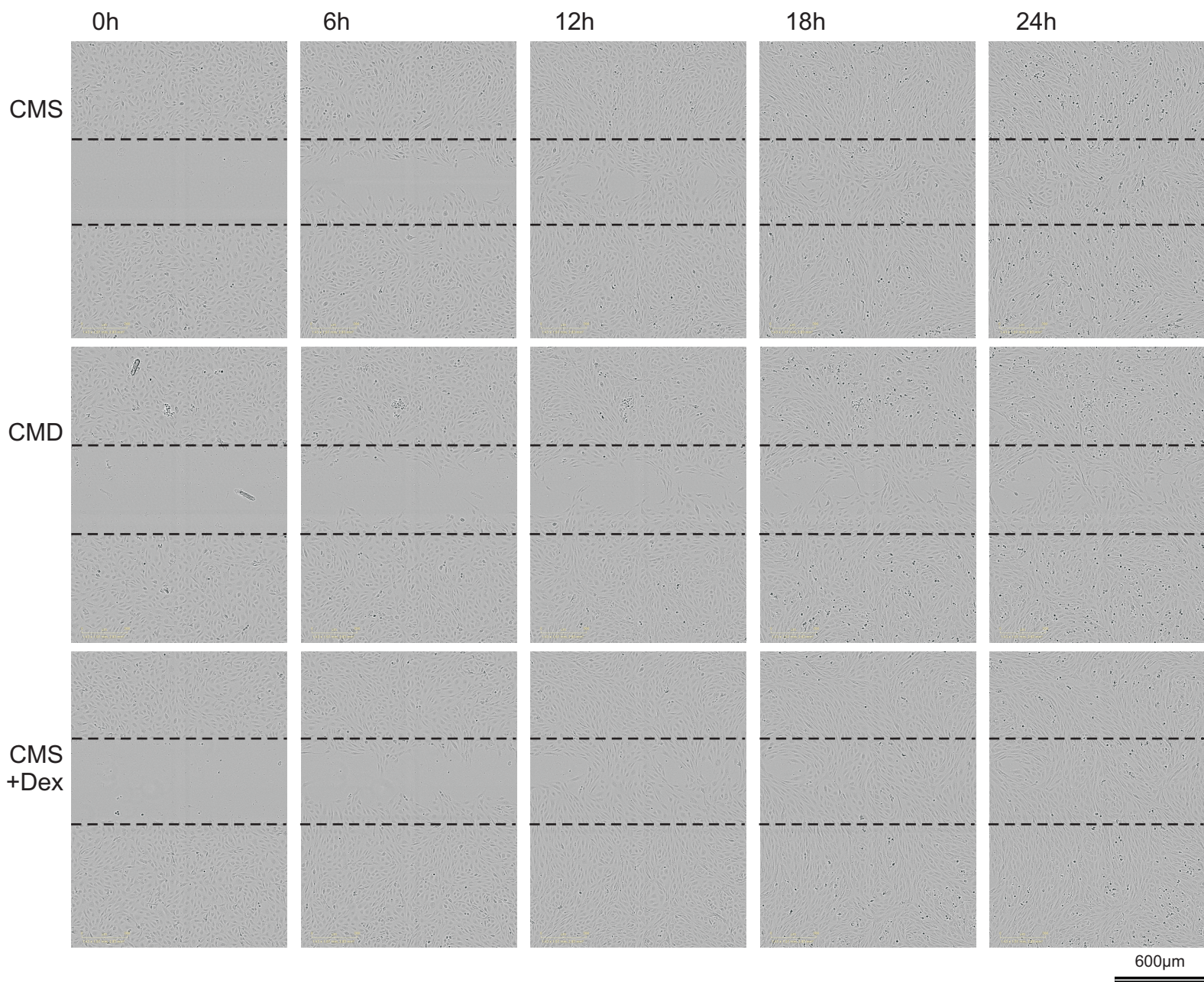

Supplement: Supplementary file 1 — Supplementary Figure 1. Expression of uPA in conditioned medium is reduced when the myofibroblasts are exposed for 6h to dexamethasone. Supplementary Figure 2. Levels of prostanoids produced in HUVECs are not affected by treatment with conditioned medium from myofibroblasts. Supplementary Figure 3. Conditioned media does not alter acetylated LDL uptake by HUVECs. Supplementary Figure 4. Representative images of scratch wound assay. [file mmc1.pdf]

ZD\_Supplementary Figure 5

A. HUVEC, 6h incubation

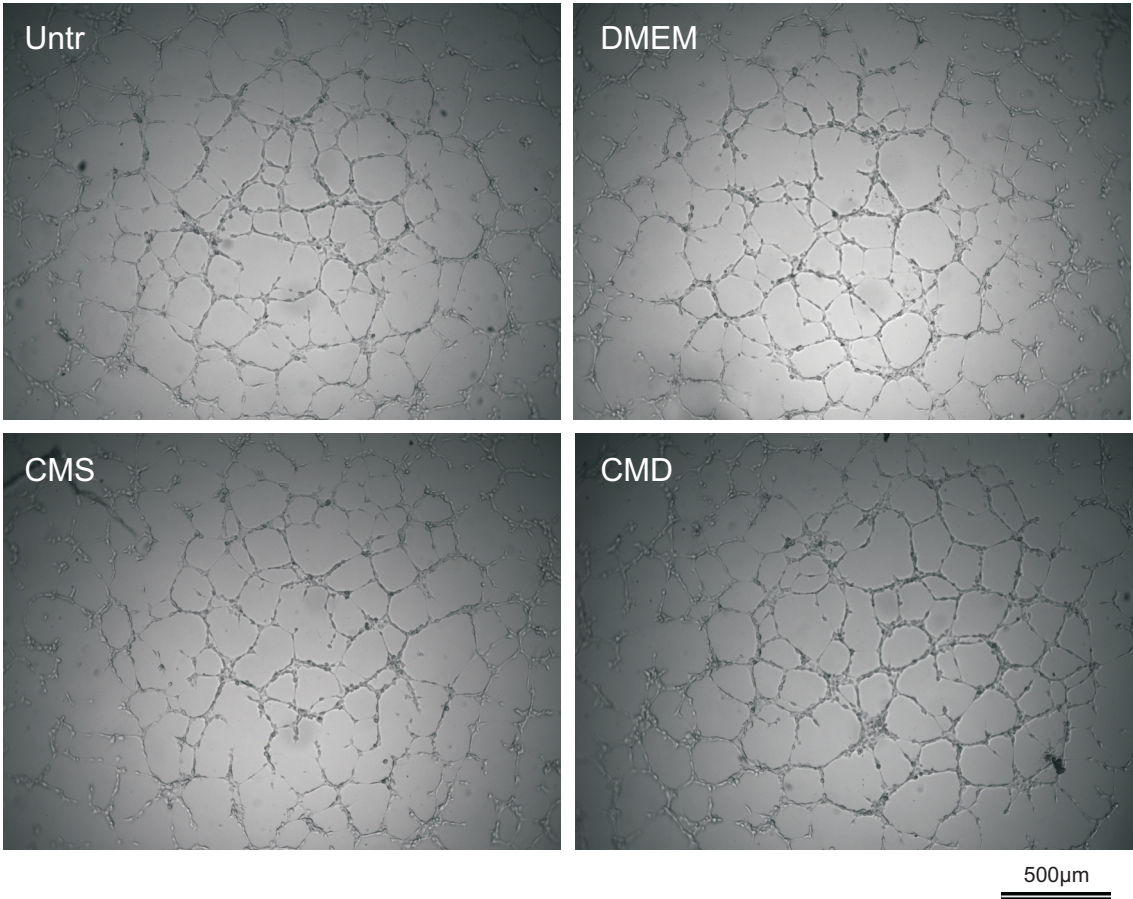

B. HAoEC, 3h incubation

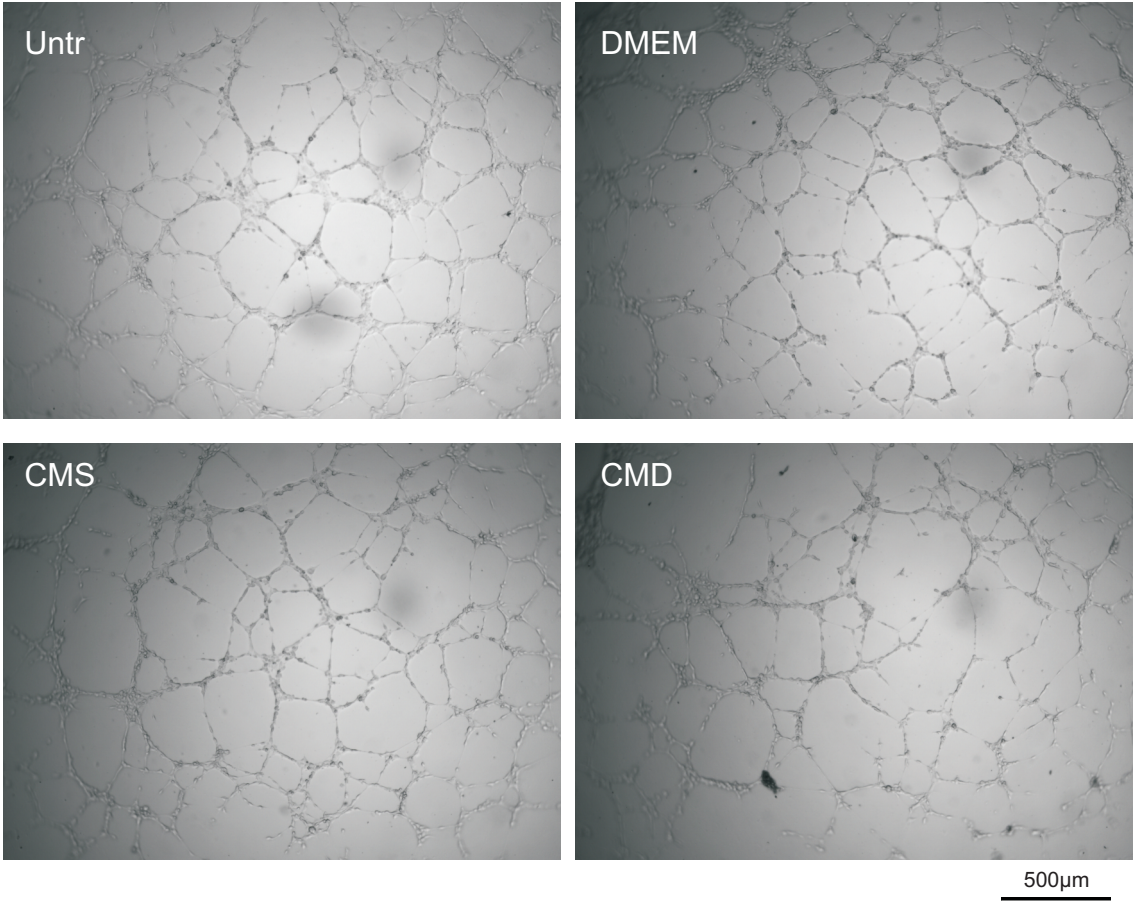

Supplement: Supplementary Figure 5 — Representative images of tube-like structure formation assay. [file mmc2.pdf]

ZD\_Supplementary Figure 6

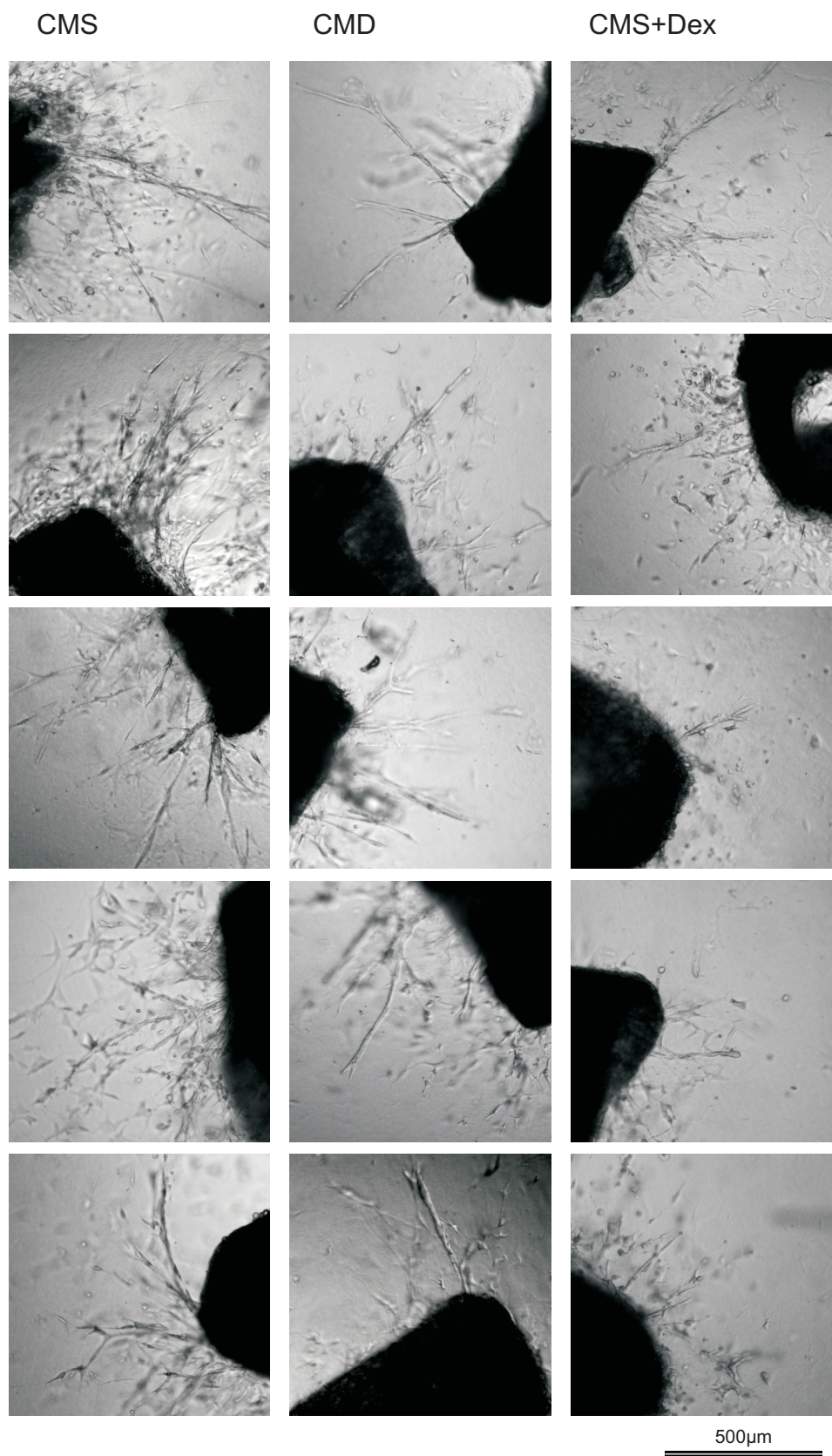

Supplement: Supplementary Figure 6 — Representative higher-power images of the aortic rings and outgrowths. [file mmc3.pdf]
