# Supplementary material for: Colon cancer-derived myofibroblasts increase endothelial cell migration by glucocorticoid-sensitive secretion of a pro-migratory factor
Source: Vascul Pharmacol. 2017 Feb;89:19–30. doi: 10.1016/j.vph.2016.10.004 (PMC5328197; doi:10.1016/j.vph.2016.10.004)
Supplement: Supplementary file 4 — Supplementary material comprises of supplementary methods, supplementary tables and legends for supplementary figures. [file mmc4.doc]

**Supporting Information**

**Supplementary methods**

*Acetylated-low density lipoprotein (Ac-LDL) uptake assay*

HUVECs were seeded onto glass coverslips (coated with 0.1% gelatin) and incubated overnight at 37°C, 5% CO_2_. Cells were then incubated for 24h in EGM2^S+^ (control), DMEM, CMD or CMD. DMEM and 10-fold concentrated CM were diluted 1:1 with EGM2^S+^. Ac-LDL uptake was assessed as described [64]. Briefly, HUVECs were incubated for 5h with 5 µg/ml Ac-LDL conjugated with alexa-488 (Invitrogen, Glasgow, UK, cat no: L23380), then washed with PBS, fixed with 2% paraformaldehyde, washed again with PBS and stained with DAPI. Images were taken using a fluorescence microscope (Axioscope, Zeiss, Oberkochen, Germany), CoolSNAP camera (Photometrics, AZ, USA) and MCID Basic 7.0 software. Photographs were analyzed for green fluorescence signal intensity over number of cells per image, using ImageJ software [33].

***Supplementary Figure 1. Expression of uPA in conditioned medium is reduced when the myofibroblasts are exposed for 6h to dexamethasone.***

CT5.3hTERT cells cultured in DMEM were treated for 6h with solvent, Dex (1µM) or CpdA (10µM). Isolated mRNA was subjected to RT-qPCR assaying uPA mRNA levels. Results were normalized to the respective geometric mean of GAPDH, PPIB and 36B4 reference genes’ mRNA levels and are expressed relative to the solvent control. Results are the mean ± SD of three independent experiments and statistical analysis was performed using a one-way analysis of variance (ANOVA) and Tukey’s multiple comparisons post-test. ns: not significant ***: p<0.001.

***Supplementary Figure 2. Levels of prostanoids produced in HUVECs are not affected by treatment with conditioned medium from myofibroblasts.***

**(A-F)** HUVECs were treated with either EGM2^S+^ or EGM2^S+^ mixtures with CMS or CMD in 1:1 ratio. After 24h cells and media were collected and analyzed for prostanoids. In HUVEC media and cell lysates ELISA was performed to quantify **(A, D)** PGF_2α_, **(B, E)** PGI_2_ (by assessing 6-keto-PGF_1α_) and **(C, E)** PGE_2_ concentrations. Results are the mean ± SD of three independent experiments and statistical analysis was performed using a one-way analysis of variance (ANOVA) and Tukey’s multiple comparisons post-test, ns: not significant.

***Supplementary Figure 3. Conditioned media does not alter acetylated LDL uptake by HUVECs.***

HUVECs were seeded onto coverslips and cultured for 24h in either EGM2^S+^ (control) or with EGM2^S+^ containing DMEM, CMS or CMD in 1:1 ratio. Ac-LDL conjugated with Alexa-488 (5 µg/ml) was added to the cells (5h) before they were washed, fixed, counterstained (DAPI) and quantified. Tukey’s box plots represent data of three independent experiments and were analyzed using a Mann-Whitney U test. ns: not significant.

*Supplementary Figure 4. Representative images of scratch wound assay.*

HUVECs were cultured in EGM2^S+^. After 18h a wound was created in the confluent cell monolayer. Cells were washed and treated with EGM2^S-^ mixtures with CMS, CMD or CMS+Dex (50 nM) in 1:1 ratio. Examination of the wound healing process and image capture were performed with the IncuCyte ZOOM system. Dotted lines indicate the position of the original scratch wound.

***Supplementary Figure 5. Representative images of tube-like structure formation assay.***

HUVECs **(A)** and HAoECs **(B)** were seeded on Matrigel-coated wells and treated with either EGM2^S+^ or EGM2^S+^ mixtures with DMEM, CMS or CMD in 1:4 ratio. Phase-contrast images were taken at 6h post induction for HUVECs and 3h post induction for HAoECs.

***Supplementary Figure 6. Representative higher-power images of the aortic rings and outgrowths.***

Explants were prepared from aortas isolated from adult male C57BL/6 mice. After embedding in collagen, aortic rings were treated with CMS, CMD or CMS+Dex (50nM), in 1:1 ratio with serum-free DMEM. Images of explants and vascular sprouts were captured after 10 days.

*Supplementary Table 1*. Conditioned medium dilutions and concentrations used in experiments

| **Figure** | **Cell Type** | **Experiment** | **Conditioned medium : EGM2** | **Conditioned medium final concentration** | **EGM2 used for dilutions** |
| --- | --- | --- | --- | --- | --- |
| 3A | HUVEC | MTT assay | 1:1 | 5x | +serum (2%) |
| 3B | HUVEC | SRB assay | 1:1 | 5x | +serum (2%) |
| 3C | HUVEC | SRB assay | 1:1 | 5x | serum-free |
| 4, suppl. fig. 4 | HUVEC | scratch assay | 1:1 | 5x | serum-free |
| 5, suppl. fig. 5 | HUVEC  HAoEC | TLS formation | 1:4 | 2x | +serum (2%) |
| 6 | HUVEC | RT-qPCR | 1:1 | 5x | +serum (2%) |
| 7, 8, suppl. fig 6. | murine aortic rings | aortic ring assay | 1:1 with DMEM | 5x | serum-free DMEM |
| suppl. fig. 2 | HUVEC | ELISA | 1:1 | 5x | +serum (2%) |
| suppl. fig. 3 | HUVEC | Ac-LDL assay | 1:1 | 5x | +serum (2%) |

***Supplementary Table 2.* A summary of factors detected or not-detected in the conditioned medium from myofibroblasts.**

| *Factors detected in CM* | *Factors not detected in CM* |
| --- | --- |
| | Angiogenin | | --- | | Angiopoietin-1  Angiostatin  ANGPTL-2  Endostatin  HGF/SF  IL-8  MCP-1  TNFβ  TSP-1  u-PA  VEGF | | | Angiopoietin-2 | | --- | | Angiopoietin-4  ANGPTL-1  bFGF  GM-CSF  IL-1α  IL-1β  IL-6  PDGF  TNFα  TSP-4  VEGFR-2 | |

***Supplementary Table 3.* List of primers used in the qPCR analysis (F- forward, R-reverse)**

| **Gene** | **Sequence (5' - 3')** | **Source** |
| --- | --- | --- |
| h36B4 | F: CATGCTCAACATCTCCCCCTTCTCC | [65] |
|  | R: GGGAAGGTGTAATCCGTCTCCACAG |  |
| hANG | F:CCGTTTCTGCGGACTTGTTC | [66] |
|  | R:GCCCATCACCATCTCTTCCA |  |
| hANGPTL2 | F:AGACGCCTGGATGGCTCTGTTA |  |
|  | R:AGTTGCCTTGGTTCGTCAGCCA |  |
| hGAPDH | F: AGCCACATCGCTCAGACAC |  |
|  | R: GCCCAATACGACCAAATCC |  |
| hIL6 | F: GACAGCCACTCACCTCTTCA |  |
|  | R: AGTGCCTCTTTGCTGCTTTC |  |
| huPA | F:CACGCAAGGGGAGATGAA | [67] |
|  | R:ACAGCATTTTGGTGGTGACTT |  |
| hVEGF | F: CACCCATGGCAGAAGGAGGA |  |
|  | R: ACACACTCCAGGCCCTCGTC |  |
| hVEGFR1 | F: CGCTTGCCAGCTACGGTTTC |  |
|  | R: GGCGACGAATTGACCAAAGC |  |
| hVEGFR2 | F: GGAACCTCACTATCCGCAGAGT |  |
|  | R: CCAAGTTCGTCTTTTCCTGGGC |  |
| m/hPPIB | F: ATGGTGATCTTCTTGCTGGTCCTTGC | [65] |
|  | R: GCATACGGGTCCTGGCATCTTGTCC |  |

**Supplementary References**

[64] J.C. Voyta, D.P. Via, C.E. Butterfield, B.R. Zetter, Identification and isolation of endothelial cells based on their increased uptake of acetylated-low density lipoprotein, J Cell Biol 99(6) (1984) 2034-40.

[65] N. Bougarne, R. Paumelle, S. Caron, N. Hennuyer, R. Mansouri, P. Gervois, B. Staels, G. Haegeman, K. De Bosscher, PPARalpha blocks glucocorticoid receptor alpha-mediated transactivation but cooperates with the activated glucocorticoid receptor alpha for transrepression on NF-kappaB, Proc Natl Acad Sci U S A 106(18) (2009) 7397-402.

[66] S. Sadagopan, N. Sharma-Walia, M.V. Veettil, V. Bottero, R. Levine, R.J. Vart, B. Chandran, Kaposi's sarcoma-associated herpesvirus upregulates angiogenin during infection of human dermal microvascular endothelial cells, which induces 45S rRNA synthesis, antiapoptosis, cell proliferation, migration, and angiogenesis, J Virol 83(7) (2009) 3342-64.

[67] R. Castello, A. Estelles, C. Vazquez, C. Falco, F. Espana, S.M. Almenar, C. Fuster, J. Aznar, Quantitative real-time reverse transcription-PCR assay for urokinase plasminogen activator, plasminogen activator inhibitor type 1, and tissue metalloproteinase inhibitor type 1 gene expressions in primary breast cancer, Clin Chem 48(8) (2002) 1288-95.
